# Ester Prodrugs of Malonate with Enhanced Intracellular Delivery Protect Against Cardiac Ischemia-Reperfusion Injury In Vivo

**DOI:** 10.1007/s10557-020-07033-6

**Published:** 2020-07-09

**Authors:** Hiran A. Prag, Laura Pala, Duvaraka Kula-Alwar, John F. Mulvey, Du Luping, Timothy E. Beach, Lee M. Booty, Andrew R. Hall, Angela Logan, Volha Sauchanka, Stuart T. Caldwell, Ellen L. Robb, Andrew M. James, Zhelong Xu, Kourosh Saeb-Parsy, Richard C. Hartley, Michael P. Murphy, Thomas Krieg

**Affiliations:** 1 MRC Mitochondrial Biology Unit, University of Cambridge, Cambridge Biomedical Campus, Cambridge CB2 0XY, UK; 2 Department of Medicine, University of Cambridge, Cambridge CB2 0QQ, UK; 3 School of Chemistry, University of Glasgow, Glasgow G12 8QQ, UK; 4 Tianjin Medical University, Tianjin 300070, China; 5 Department of Surgery, University of Cambridge and NIHR Cambridge Biomedical Research Centre, Cambridge CB2 0QQ, UK

**Keywords:** Ischemia/reperfusion injury, Succinate, Malonate, Mitochondria, Drug delivery

## Abstract

**Purpose:**

Mitochondrial reactive oxygen species (ROS) production upon reperfusion of ischemic tissue initiates the ischemia/reperfusion (I/R) injury associated with heart attack. During ischemia, succinate accumulates and its oxidation upon reperfusion by succinate dehydrogenase (SDH) drives ROS production. Inhibition of succinate accumulation and/or oxidation by dimethyl malonate (DMM), a cell permeable prodrug of the SDH inhibitor malonate, can decrease I/R injury. However, DMM is hydrolysed slowly, requiring administration to the heart prior to ischemia, precluding its administration to patients at the point of reperfusion, for example at the same time as unblocking a coronary artery following a heart attack. To accelerate malonate delivery, here we developed more rapidly hydrolysable malonate esters.

**Methods:**

We synthesised a series of malonate esters and assessed their uptake and hydrolysis by isolated mitochondria, C2C12 cells and in mice in vivo. In addition, we assessed protection against cardiac I/R injury by the esters using an in vivo mouse model of acute myocardial infarction.

**Results:**

We found that the diacetoxymethyl malonate diester (MAM) most rapidly delivered large amounts of malonate to cells in vivo. Furthermore, MAM could inhibit mitochondrial ROS production from succinate oxidation and was protective against I/R injury in vivo when added at reperfusion.

**Conclusions:**

The rapidly hydrolysed malonate prodrug MAM can protect against cardiac I/R injury in a clinically relevant mouse model.

## Introduction

**Fig. 1:**
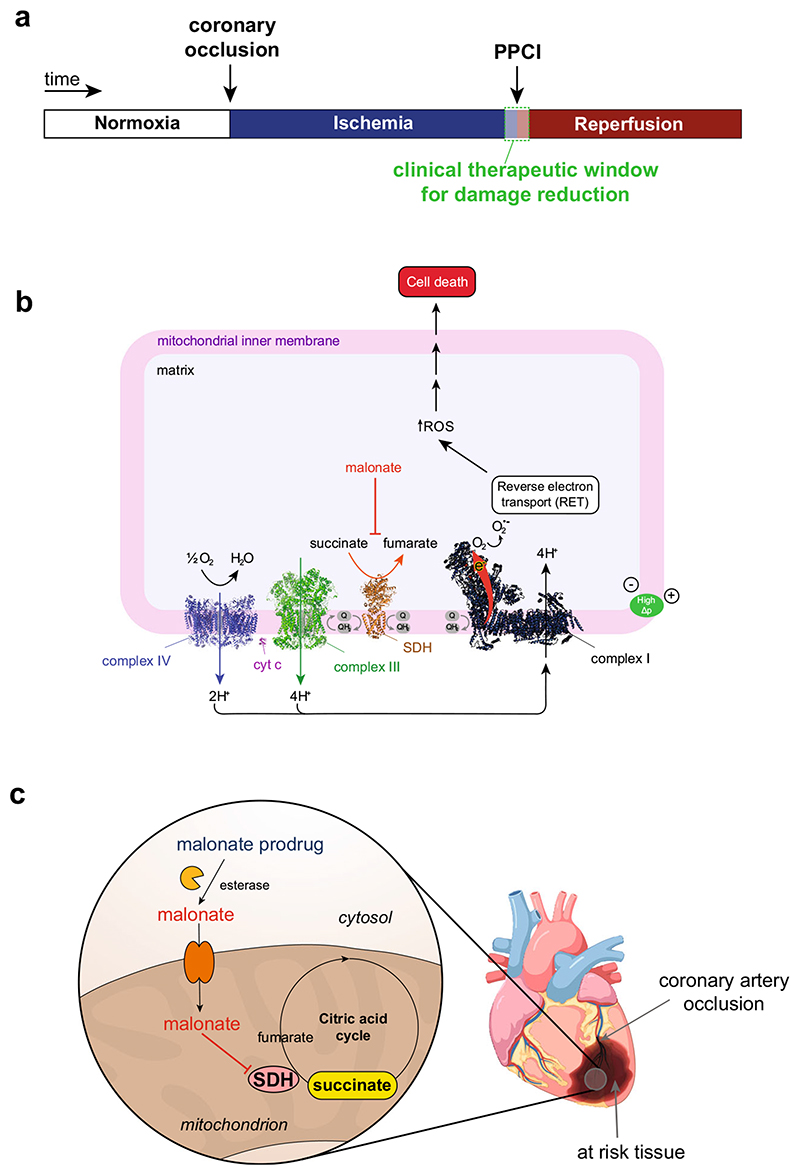
Mechanism of I/R injury and requirements for a malonate drug. **a** Timeline of clinically relevant therapeutic window in MI patients undergoing PPCI. **b** During ischemia, succinate accumulates. Upon reperfusion, succinate is rapidly oxidised by SDH allowing proton pumping from complex III and IV, maintaining the highly reduced CoQ pool and generating a large Δp, thus driving electrons through complex I by RET. RET transfers electrons to oxygen, generating superoxide initiating the oxidative damage in I/R injury. **c** Malonate prodrugs could be administered during PPCI where they would enter cells, hydrolyse to release malonate and go on to inhibit SDH in mitochondria

Myocardial infarction (MI) is a leading cause of mortality in the developed world [1, 2]. MIs occur due to a blockage in the coronary blood supply, rendering the tissue ischemic that leads on to cardiomyocyte death [2–6]. Rapid reperfusion of the ischemic myocardium by removing the coronary artery blockage, typically by primary percutaneous coronary intervention (PPCI), is the standard of care for MI treatment [5, 6] (Fig. 1a). However, paradoxically, the reintroduction of oxygen to the ischemic tissue upon reperfusion causes significant damage to the heart [4, 7, 8]. This damage is characterised by a burst of mitochondrial ROS production that initiates the train of events that leads to cardiomyocyte death by ischemia/reperfusion (I/R) injury [9–11]. I/R injury is a critical driver for the development of post-MI heart failure and as there are no current therapies, presents a significant unmet need [2, 7, 12, 13].

Until recently, the mitochondrial ROS production that initiates I/R injury was thought to occur relatively randomly as part of the chaotic series of events associated with the reperfusion of ischemic tissue. It is now apparent that during ischemia, the mitochondrial metabolite succinate accumulates selectively [9, 10, 14]. This rewiring of the citric acid cycle (CAC) occurs in response to ischemia and the associated purine nucleotide breakdown. During ischemia, the electrons that accumulate on NADH in the cytosol reduce oxaloacetate to malate, which is transported into mitochondria, where it generates fumarate and NADH [10, 11]. This causes extensive reduction of the mitochondrial CoenzymeQ (CoQ) pool which can both drive the reversal of succinate dehydrogenase (SDH), thereby reducing fumarate to succinate, and also prevent the oxidation of succinate generated by the CAC [14]. Succinate accumulation within tissues, along with purine nucleotide loss, are now seen as hallmarks of ischemia in a range of tissues and species [10, 15–17]. Upon reperfusion, the reintroduced oxygen rapidly oxidises the accumulated succinate [10, 18], restoring proton pumping by respiratory complexes III and IV. This generates a highly reduced CoQ pool and a large proton motive force (Δp) across the mitochondrial inner membrane which is close to maximal because the low levels of ADP curtail its consumption by ATP synthesis [17, 19]. Together, these drive the production of ROS by reverse electron transport (RET) through complex I [10, 20] (Fig. 1b). RET seems to be the principal source of the ROS burst during the first minutes of reperfusion that initiates I/R injury in MI [10, 11, 21].

Due to its essential role in I/R injury, therapeutic strategies targeting both succinate accumulation and/or its oxidation have potential [21, 22]. This is supported by the efficacy of the cell-permeable dimethyl malonate (DMM). DMM is a prodrug of the SDH competitive inhibitor malonate which prevents both succinate accumulation and oxidation, and thereby decreases cardiac I/R injury [10, 17, 23]. As malonate is both a reversible inhibitor of SDH and an endogenous molecule that is readily metabolised, it has excellent therapeutic attributes. However, to be clinically effective in treating MI, the malonate prodrug must be administered upon reperfusion during PPCI (Fig. 1a) and be both taken up and hydrolysed to malonate within a few minutes (Fig. 1c). Whilst the disodium salt of malonate itself has shown to be cardioprotective in a large animal model, the mechanism of uptake into cardiomyocytes is unclear and the requirement to use high concentrations injected intracoronary may limit its use [15]. At physiological pH, malonate is largely in its dianionic form (pKa_1_ =2.83, pKa_2_ = 5.69 [24]); therefore, the proportion of neutral malonate, capable of diffusion across biological membranes, is negligible at physiological pH (< 0.003% at pH 7.4). Thus, the unmediated diffusion of malonate across biological membranes is low. Therefore, to enhance the acute delivery of malonate to the heart during PPCI, we set out to assess a series of malonate esters, thereby masking the charged moieties and creating a neutral and membrane permeable malonate prodrug [25]. The malonate prodrugs would subsequently be hydrolysed in the cell to release malonate rapidly in vivo, to inhibit SDH activity and prevent I/R injury (Fig. 1c).

## Materials and Methods

### Chemical Syntheses

The fluorinated esters ditrifluoroethyl malonate (MTF) and dihexafluoropropyl malonate (MHF) were synthesised by reaction of malonyl chloride with excess trifluoroethanol or 1,1,1,3,3,3-hexafluoro-2-propanol, respectively. Diacetoxymethyl malonate (MAM) was prepared by O-alkylation of malonate with acetoxymethyl bromide following the procedure of Bao et al. [26]. The details of the chemical syntheses are described in the “Electronic Supplementary Material” section.

### Maintenance of Cells in Culture

C2C12 (murine) and HeLa (human) cells were obtained from American Type Culture Collection (ATCC). Both were maintained at 37 °C, 5% CO_2_ and 100% humidity. Cell media was changed every 2–3 days with passaging at < 80% confluency and seeding at no higher than 5 × 10^3^ viable cells/cm^2^ with a typical subcultivation ratio of 1:5–1:10. Cell media was DMEM media (4.5 g/l glucose, 1 mM sodium pyruvate, 2 mM Glutamax, 1.5 g/l sodium bicarbonate) with 10% Foetal Bovine Serum (FBS), 100 U/ml penicillin and 100 mg/ml streptomycin.

### Animals

Procedures were carried out in accordance with the UK Animals (Scientific Procedures) Act of 1986 and the University of Cambridge Animal Welfare Policy under project licence 70/8238 and 70/7963, reviewed by the University of Cambridge Animal Welfare Ethical Review Board or the Tianjin Medical University Animal Care and Use Committee. All animal experiments described were carried out in accordance with the Guide for the Care and Use of Laboratory Animals, published by the US National Institutes of Health (NIH Publication No. 85-23, revised 1996). Wistar rats (female, 10–12 weeks) and C57BL/6 J mice (male, 8–12 weeks) were ordered from Charles River Laboratories UK (Margate, UK) or the Institute of Laboratory Animal Science, Chinese Academy of Medical Sciences (Beijing, China). Both were maintained with ad libitum chow and water.

### LC-MS/MS Analysis of Malonate and Succinate

LC-MS/MS analysis of malonate and succinate was performed using an LCMS-8060 mass spectrometer (Shimadzu, UK) with a Nexera UHPLC system (Shimadzu, UK). Samples were stored in a refrigerated autosampler (4 °C) upon injection of 5 μl into a 15 μl flowthrough needle. Separation was achieved using a SeQuant ZIC-HILIC column (3.5 μm, 100 Å, 150 × 2.1 mm, 30 °C column temperature; Merck Millipore, UK) with a ZIC-HILIC guard column (200 Å, 1 × 5 mm). A flow rate of 200 μl/min was used with mobile phases of: A) 10 mM ammonium bicarbonate (pH unchanged); and B) 100% acetonitrile. A gradient of 00.1 min, 80% MS buffer B; 0.1–4 min, 80–20% B; 4–10 min, 20% B; 10–11 min, 20–80% B; and 11–15 min, 80% B was used. The mass spectrometer was operated in negative ion mode with multiple reaction monitoring (MRM), and spectra were acquired using the LabSolutions software (Shimadzu, UK), with compound quantities calculated from relevant standard curves in MS extraction buffer (50% (v/v) methanol, 30% (v/v) acetonitrile, 20% (v/v) MS-grade water) compared with 1 nmol of relevant internal standard either [^13^C_3_]-malonate or [^13^C_4_]-succinate for malonate and succinate, respectively (Supplementary Fig. 1).

### Hydrolysis of Malonate Esters

Malonate esters (200 μM) were incubated in KCl buffer (120 mM KCl, 10 mM HEPES, 1 mM EGTA pH 7.2 or 8, 37 °C)pH 7.2 or 8, 37 °C) alone or KCl buffer supplemented with porcine liver esterase (1 mg protein/ml; pH 7.2, 37 °C; PLE) on a heated shaking block (37 °C, 1000 rpm)1. Aliquots of 20 μl were taken at time points of 0, 15, 30 min, 1, 2 and 4 h. Additional time points were taken in PLE-free incubations, at 8 and 24 h. Samples were extracted in 750 μl MS extraction buffer containing MS internal standard (1 nmol) and analysed by LC-MS/MS. Importantly, controls showed that there was no hydrolysis of these esters when directly analysed by LC-MS/MS, thus malonate release was solely from hydrolysis under the incubation conditions (data not shown).

### Isolation of Rat Heart Mitochondria

Rat heart mitochondria (RHM) were isolated as described previously [27]. Briefly, rats were culled by cervical dislocation in accordance with local procedures; the heart rapidly excised and washed with STEB buffer (250 mM sucrose, 5 mM Tris-Cl, 1 mM EGTA, 0.1% (w/v) BSA; pH 7.4, 4 °C) and minced finely. The minced tissue was homogenised in a dounce homogeniser in the buffer and centrifuged (1000 ×g, 5 min, 4 °C). The resulting supernatant was filtered through pre-wet muslin and centrifuged to pellet mitochondria (10,000 ×g, 10 min, 4 °C). The mitochondrial pellet was resuspended in buffer and re-centrifuged under the same conditions to pellet mitochondria. The final mitochondrial pellet for all tissues was resuspended in STE buffer (no BSA) and assayed for protein concentration by BCA assay (Thermo Fisher Scientific, UK).

### Preparation of Bovine Heart Mitochondrial Membranes

Bovine heart mitochondria (BHMMs) were isolated by differential centrifugation similarly to rat heart mitochondria and as described previously [28]. To prepare (BHMMs), bovine heart mitochondria were blended with MilliQ water (4 °C) before adding KCl (150 mM final concentration) and continuing to blend until homogeneous. The suspension was centrifuged (13,500 ×g, 40 min, 4 °C), the supernatant discarded and the pellet homogenised in resuspension buffer (20 mM Tris-Cl, 1 mM EDTA, 10% glycerol, pH 7.55 at 4 °C) before snap freezing membranes and storing at - 80 °C until use. The protein concentration of the membranes was determined by BCA assay (Thermo Fisher Scientific, UK).

### Complex II + III Activity Assay

BHMMs (80 μg protein/ml) were incubated in potassium phosphate buffer (50 mM potassium phosphate, 1 mM EDTA, pH 7.4, 4 °C) supplemented with KCN (3 mM), rotenone (4 μg/ml) and succinate (as indicated). In a 96-well microplate, malonate or vehicle control and membrane incubations were plated and incubated (10 min, 30 °C). Oxidised cytochrome c was added prior to measuring respiratory chain activity by following the reduction of cytochrome c spectrophotometrically at 550 nm (20-s intervals for 5 min, 30 °C; Spectramax Plus 384, Molecular Devices, UK). Final concentrations of bovine heart mitochondrial membranes (10 μg protein/well), cytochrome c (30 μM) and succinate (either 0.2, 1 or 5 mM) were used. Control complex II+III activity with 200 μM, 1 mM or 5 mM succinate was 154.6 ± 3.7, 268.2 ± 4.6, 345.9 ± 8.7 nmol Cyt c reduced/min/mg protein, respectively.

### Malonate Delivery to Mitochondria

Isolated mitochondria (0.5 mg protein/ml) were incubated in KCl buffer (pH 7.4; 37 °C) supplemented with glutamate (1 mM) and malate (1 mM) and dipotassium malonate or malonate esters (200 μM) for 5 min before pelleting mitochondria (10,000×g, 5 min, 4 °C), rinsing the tube without disturbing the pellet before drying the pellet by aspirating and blotting residual liquid with tissue paper. Pellets were extracted with MS extraction buffer containing MS internal standard and analysed by LC-MS/MS. To calculate the occluded malonate in the mitochondrial pellet that was not within the mitochondria (for dipotassisum malonate incubations), we used a value obtained earlier in our lab for the non-mitochondrial volume determined by comparing the [^14^C]-sucrose and H_2_O volumes of the pellet [29]. This value was 8.1 μl/mg protein. The concentration of malonate within the mitochondria was calculated using a mitochondrial volume of 0.6 μl/mg protein [29].

### Measurement of ROS Production by RET

ROS production by RET in isolated heart mitochondria was measured by following the conversion of Amplex Red to resorufin as described previously [27]. Briefly, in a 96-well plate, isolated RHM were incubated in 150 μl KCl buffer (pH 7.2, 37 °C) supplemented with Amplex Red (Invitrogen, Thermo Fisher Scientific), horseradish peroxidase, BSA, superoxide dismutase and either malonate, malonate diesters or vehicle control. To initiate RET, 50 μl KCl supplemented with succinate was added to each well and the plate read using a fluorometric plate reader (SpectraMax Gemini XS; Molecular Devices) at 37 °C. Resorufin fluorescence was detected by λ_ex_ = 570 nm and λ_em_ = 585 and calibrated against known concentrations of hydrogen peroxide (46.6 M^–1^ cm^–1^ at 240 nm). The final concentrations in the assay were as follows: RHM (70 μg protein/well), Amplex Red (12.5 μM), horseradish peroxidase (20 μg/ml), BSA (200 μg/ml), superoxide dismutase (40 μg/ml), malonate (0–5 mM), malonate diesters (250 μM) and succinate (0–5 mM or 5 mM when used with malonate diesters).

### Incubation of Cells with Malonate Diesters

C2C12 or HeLa cells were plated in 6-well plates (300,000 cells/well) and adhered overnight. The following day, the medium was replaced with Krebs buffer (116 mM NaCl, 4.7 mM KCl 1.2 mM MgSO_4_.7H_2_O, 25 mM NaHCO_3_ 1.4 mM CaCl_2_, 11 mM glucose; pH 7.4, 37 °C) containing malonate diesters or 0.1% DMSO (control) and incubated for 15–240 min at 37 °C prior to extracting. Parallel plates were incubated under the same conditions and used to measure protein levels by BCA assay (Thermo Fisher Scientific, UK). After incubation, cells and supernatant were extracted for LC-MS/MS. The supernatant was removed and centrifuged (17,000× g, 10 min, 4 °C) before adding 50 μl of the resulting supernatant to 750 μl MS extraction buffer containing MS internal standard. Cells were washed 4 times with ice-cold PBS before 500 μl MS extraction buffer containing MS internal standard was added to each well and incubated for 15 min on dry ice. Cells were scraped into microcentrifuge tubes and together with the supernatant samples were agitated (1200 rpm, 15 min, 4 °C) before incubating at - 20 °C for 1 h. Samples were centrifuged (17,000 ×g, 10 min, 4 °C); the supernatant transferred to a fresh tube and recentrifuged under the same conditions. The resulting supernatant was transferred to precooled MS vials and analysed by LC-MS/MS.

### In Vivo Delivery of Malonate Diesters

DMM, MAM or MTF (160 mg/kg) were administered to C57BL/6 J mice via a 100 μl tail vein bolus injection in 0.9% saline + 0.1% DMSO. Mice were culled either 5, 20 or 40 min after injection, organs harvested and snap frozen in liquid nitrogen. Malonate was extracted from tissues by homogenising tissue (40 mg wet weight/ml) in MS extraction buffer containing MS internal standard in a Precellys 24 tissue lyser (6500 rpm, 15 s × 2; Bertin Instruments, France) using CK-28R homogenising tubes (Bertin Instruments, France), then the homogenised samples were incubated (- 20 °C, 1 h). Samples were centrifuged (17,000 ×g, 10 min, 4 °C); the supernatant transferred to a fresh tube and recentrifuged under the same conditions. The resulting supernatant was transferred to pre-cooled MS vials and analysed by LC-MS/MS.

### In Vivo Left Anterior, Descending Coronary Artery (LAD) Occlusion Murine Myocardial Infarction Model

The left anterior descending (LAD) coronary artery was ligated to induce MI in an open chest, in situ mouse model as previously described, to assess the effects of compounds on I/R injury [30]. Briefly, mice were anesthetised by administration of sodium pentobarbital (70 mg/kg intraperitoneally), endotracheally intubated, ventilated with 3 cm H_2_O positive end expiratory pressure and kept at 37 °C using a rectal thermometer-controlled heatpad (TCAT-2LV, Physitemp, USA). Ventilation frequency was maintained at 110 breaths/min, with tidal volume between 125 and 150 μl. The heart was exposed and a suture was placed around the prominent branch of the LAD and passed through a small plastic tube used to initiate ischemia by pressing the tube against the heart surface to occlude the LAD. Mice were subjected to 30 min of ischemia and 120 min of reperfusion; after reperfusion, hearts were stained with Evans Blue and 2% triphenyltetrazolium chloride (TTC) and blindly analysed by an independent researcher.

### Statistical Analysis, Randomisation and Blinding

All data in figures are presented as mean ± S.E.M., unless stated otherwise in the figure legend. Statistical analysis was performed using either one or two-way ANOVA with the suitable post hoc correction for multiple comparisons described in the figure legend. A *p* value of less than 0.05 was considered significant. Statistics were calculated in the Prism 8.0 software (GraphPad Software Inc., USA). Randomisation and blinding were carried out where possible: mass spectrometry samples were randomised and analysed blindly; infarct size measurements were carried out in a randomised and blinded fashion by independent investigators.

## Results

### DMM Is Not Cardioprotective when Administered at Reperfusion

**Fig. 2:**
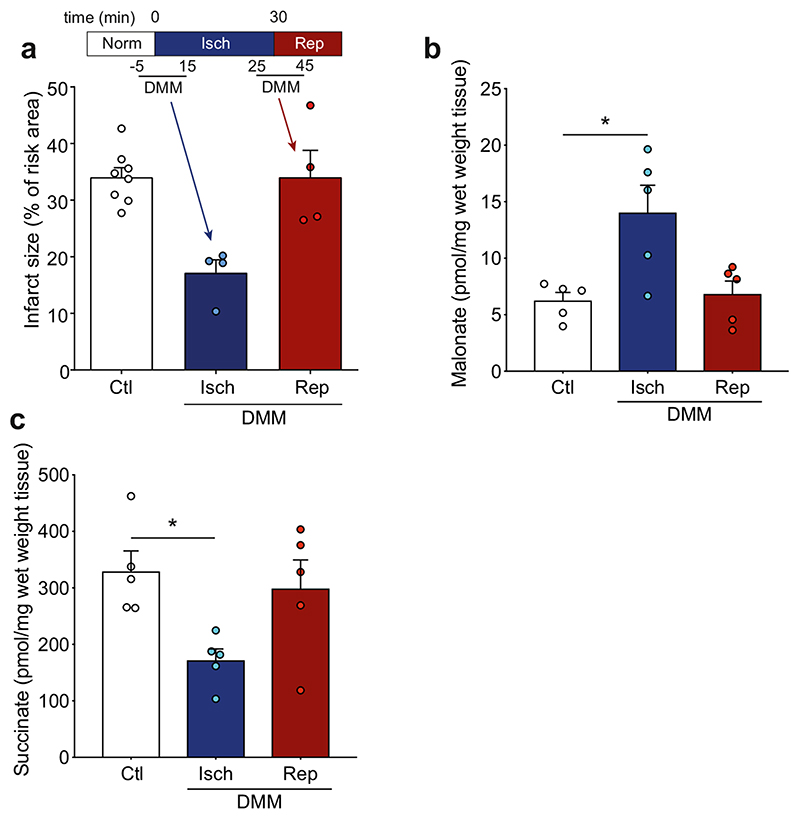
DMM is protective against I/R injury when administered during ischemia but not upon reperfusion. **a** Protection against I/R injury by DMM. C57BL/6 J mice were subjected to 30 min LAD occlusion before 2 h reperfusion and infarct assessed by TTC staining. 0.9% saline (Ctl) or DMM (160 mg/kg total dose) dissolved in 0.9% saline were administered i.v. either before ischemia or at reperfusion (mean ± SEM, *n* = 4–8). **b**-**c** Malonate levels (**b**) and succinate levels (**c**) in risk tissue at start of reperfusion. Mice were subjected to LAD occlusion as in (**a**) and DMM similarly infused but risk tissue harvested after 1 min reperfusion and malonate and succinate measured by LC-MS/MS (mean ± SEM, *n* = 5). Statistical significance was assessed by one-way ANOVA (compared with control) with Dunnett’s correction for multiple comparisons where *p < 0.05 and ****p < 0.0001

Whilst DMM has shown protection against cardiac I/R injury when administered prior to ischemia [10, 23], its protective effect when infused at reperfusion is unknown. As administration of DMM at reperfusion is more clinically relevant for myocardial infarction patients undergoing PPCI, we first determined cardioprotection by DMM when given before ischemia against DMM administration at reperfusion. This was done by measuring infarct size in the in vivo LAD mouse model of acute myocardial infarction. DMM conferred considerable cardioprotection when the infusion began shortly before ischemia but was not protective when administered prior to reperfusion (Fig. 2a and Supplementary Fig. 2). To confirm whether this was due to differences in malonate delivery, we measured the levels of malonate in the at-risk tissue and found that the levels of malonate were significantly elevated when DMM was administered prior to and during ischemia, but not when given immediately before reperfusion (Fig. 2b). The levels of succinate measured at 1 min reperfusion were also significantly lower when DMM was infused prior to and during ischemia (Fig. 2c). This suggests that the malonate released from DMM during ischemia decreases the accumulation of succinate during ischemia, as shown previously [10]. If malonate is administered on reperfusion, it would be expected to block succinate oxidation, leading to an elevation in succinate; however, the succinate levels when DMM was infused at reperfusion were not different from saline infused hearts (Fig. 2c). This suggested that the release of malonate from DMM was too slow to be protective when administered upon reperfusion.

### Development of Rapidly Cleaved Malonate Prodrugs

**Fig. 3:**
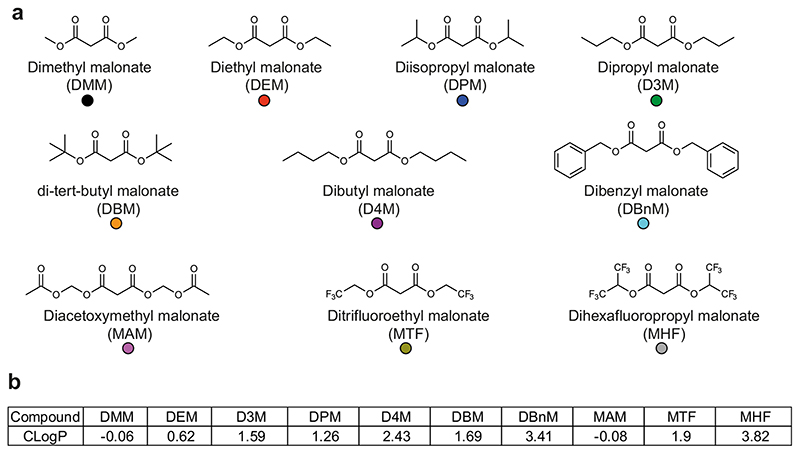
Structures of malonate prodrugs. **a** Structures of malonate prodrugs and colour identifiers used throughout. **b** Calculated Log P (CLogP) values for the compounds (calculated in ChemDraw Professional 17.1)

We next designed and assessed nine further malonate ester prodrugs with the goal of accelerating malonate delivery in vivo. Two approaches were taken to improve malonate delivery—either increasing the hydrophobicity of the molecule to enhance diffusion across biological membranes or creating esters which are hydrolysed faster by cellular esterases. A range of ester chain lengths with differing hydrophobicities were introduced, and the branched chain tert-butyl group was included as an ester resistant to enzymatic hydrolysis [31, 32]. To release malonate faster (referred to as tuned malonate esters), a preferential esterase substrate in the form of acetoxymethyl (diacetoxymethyl malonate; MAM) or electron withdrawing, fluorine-containing groups (ditrifluoroethyl malonate; MTF, and dihexafluoropropyl malonate; MHF) were introduced. The compounds are shown in Fig. 3a and their calculated octanol:water partition coefficients (CLogP) are shown in Fig. 3b. Their syntheses are described in the “Electronic Supplementary Material” section.

### Tuned Malonate Esters Rapidly Release Malonate

**Fig. 4:**
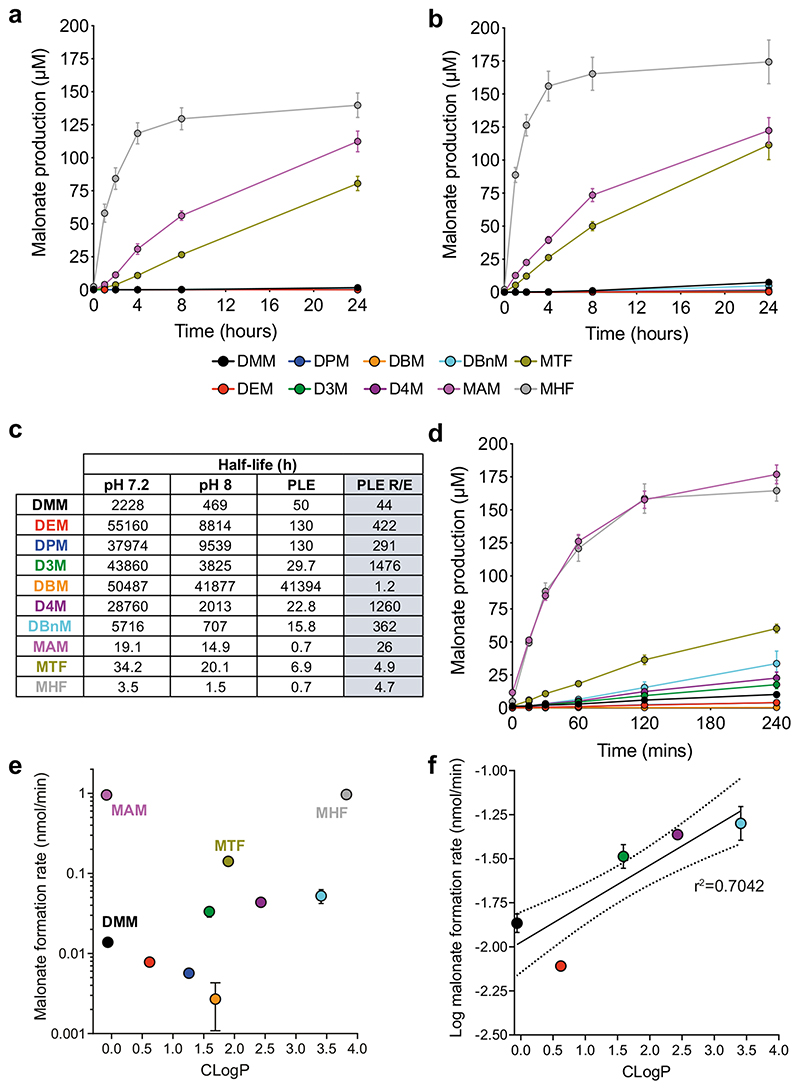
Improved malonate prodrugs are hydrolysed faster. Malonate diesters (200 μM) were incubated at 37 °C in pH 7.2 (**a** and **d**) or pH 8 (**b**) and released malonate measured by LC-MS/MS at various times. **c** Half-lives of malonate esters under incubation conditions and rate enhancement (R/E) by PLE. **d** Malonate esters were incubated as in (**a**) but porcine liver esterase (1 mg protein/ml) was added for enzymatic hydrolysis (**a**-**d** all mean ± SEM, *n* = 3). **e**, **f** Hydrolysis rate (+ PLE) correlates with CLogP in dialkyl esters from hydrolysis data in (**d**)

We first measured malonate release by spontaneous hydrolysis of the esters at the pH of the cytosol (7.2; Fig. 4a) and at the pH of the mitochondrial matrix (8; Fig. 4b). As expected, the esters with electron-withdrawing substituents (MHF, MAM and MTF) released malonate far more rapidly than DMM and this release was faster at pH 8 (Fig. 4 a and b) [33, 34]. This is consistent with both the B_Ac_2 and the E1_CB_ basecatalysed hydrolysis mechanisms that are possible [35]. To facilitate comparison of the relative hydrolysis rates of these esters, we calculated half-lives, based on malonate production which requires hydrolysis of both ester bonds in the diesters. This approach conflates two hydrolysis rates, with that for lysis of the ester bond of intermediate monoesters expected to be slower due to their negative charge. Most malonate esters were relatively stable under non-enzymatic conditions with half-lives exceeding 24 h at pH 7.2. Unsurprisingly, the electron-rich dialkyl esters hydrolysed most slowly with the half-life shortest for DMM and the longest for DBM, reflecting the anticipated steric properties of the side chains and their propensity to impact hydrolysis (Fig. 4c) [33]. Despite their increased lability, the electron poor malonate esters (MHF, MAM and MTF) were relatively stable under non-enzymatic conditions at pH 7.2: MTF’s half-life exceeded 24 h and that of MAM was ~ 19 h, but MHF’s half-life was modest at only 3.5 h (Fig. 4c). Thus, with the possible exception of MHF, all the esters are stable enough to survive in the circulation at physiological pH values for the time required for suitable cardiac intervention.

Within the cell, the expectation is that these esters will be hydrolysed by intracellular esterases. To assess this, we used porcine liver esterase (PLE), which is widely used as a model enzyme for in vivo ester hydrolysis [36–39]. As expected, all the tuned esters released malonate far more rapidly when incubated in the presence of PLE (Fig. 4d). The rate enhancement at pH 7.2 caused by PLE is shown in Fig. 4c. This analysis reveals that PLE enhanced the hydrolysis of most of the malonate esters between ~4–1500-fold. However, for DBM, there was no enhancement due to the steric hindrance of the tert-butyl groups. For MHF, the enhancement was only 4.7, due to rapid spontaneous hydrolysis and the limited effect on rate of electron withdrawing groups on the alkoxy substituent because the rate determining step in PLE is after transesterification to the enzyme [34]. The similar rate enhancement for MTF is consistent with this. Finally, we plotted the enzymatic rate of hydrolysis of these esters against their hydrophobicity (Fig. 4 e and f). This indicated that for straight chain, unfluorinated dialkyl esters increased hydrophobicity correlated with increased susceptibility to enzymatic hydrolysis.

Overall, these data confirmed that malonate release from DMM is occurring too slowly to be cardioprotective when administered at reperfusion. It also showed potential candidate compounds for further testing with enhanced hydrolysis (e.g. MAM) or with both enhanced hydrolysis and hydrophobicity (MHF and MTF).

### Malonate Ester Prodrugs Deliver Malonate to Mitochondria

**Fig. 5:**
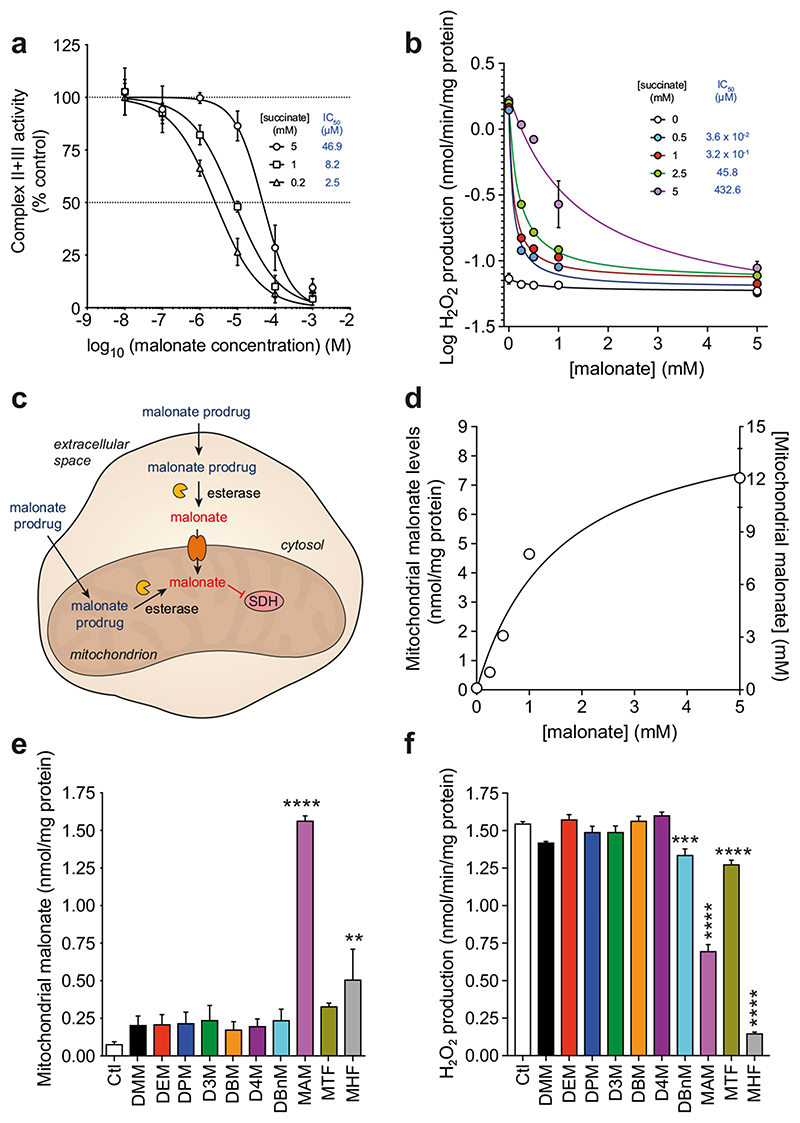
Improved malonate prodrugs deliver malonate to mitochondria. **a** SDH inhibition by malonate in bovine heart mitochondrial membranes using a complex II + III assay (mean ± SEM, *n* = 3). **b** Inhibition of ROS production by RET by malonate in isolated rat heart mitochondria (mean ± SEM, *n* = 3). **c** Schematic of cellular handling of malonate ester prodrugs and potential for malonate delivery. **d** Uptake of malonate in rat heart mitochondria. RHM (0.5 mg protein/ml) were incubated ± malonate for 5 min, before pelleting, rinsing and measuring malonate levels (mean ± SEM, *n* = 3) and a mitochondrial concentration subsequently calculated. **e** Malonate delivery from malonate esters in RHM. RHM were incubated as in (**d**) but with malonate esters (250 μM) or 0.1% DMSO (mean ± SEM, *n* = 3). **f** Inhibition of ROS production by malonate esters in RHM. RHM were incubated with succinate and malonate esters and ROS production by RET measured by the conversion of Amplex red to resorufin (mean ± SEM, *n* = 3). Statistical significance was assessed by one-way ANOVA, with Dunnett’s correction for multiple comparisons (against control values (Ctl)) where **p < 0.01, ***p < 0.001, ****p < 0.0001

We next estimated the concentration of malonate required within the ischemic tissue to prevent ROS production by succinate-driven RET. As malonate is a competitive inhibitor of SDH, the concentration required will also depend on that of succinate, which is between 1.5 and 3 mM in the ischemic mouse heart at the onset of reperfusion [40]. For mitochondrial membranes respiring on 5 mM succinate, ~ 50 μM malonate was required to inhibit SDH activity by 50% (Fig. 5a). In isolated heart mitochondria respiring on 5 mM succinate, ~ 450 μM malonate was required to inhibit by 50% ROS production by RET (Fig. 5b). The higher concentration required in isolated mitochondria likely reflects the requirement for transport of malonate into the matrix. As similar transport will be required within the tissue, this suggests that a target concentration of ~0.5 mM malonate within the cytosol is required to achieve a significant decrease in ROS production by SDH inhibition. Considering the intracellular water volume of a rodent heart is ~ 615 μl/g wet weight tissue [41], this suggests a target tissue malonate level to be ~ 0.3 nmol/mg wet weight.

The intended target of malonate for amelioration of I/R injury is SDH within mitochondria. Therefore, the handling of the malonate prodrug by the cell could occur in two ways—either hydrolysis and release of malonate within the cytosol or diffusion into mitochondria and release there (Fig. 5c). To see if malonate released in the cytosol by hydrolysis of the prodrugs would be rapidly transported into mitochondria, subsequently inhibiting SDH, we incubated mitochondria with malonate for 5 min and showed that this led to extensive uptake into mitochondria (Fig. 5d).

Thus, generating malonate in the cytosol is sufficient to deliver it to the mitochondria in the cell. To test whether malonate esters could be hydrolysed within mitochondria to deliver malonate directly, we incubated rat heart mitochondria with malonate esters and found that malonate was delivered to mitochondria by the malonate ester prodrugs (Fig. 5e). As there is negligible spontaneous hydrolysis of these esters over 5 min (Fig. 4a), this is all due to hydrolysis of the ester within the mitochondrial matrix. MAM delivered significantly more malonate to mitochondria than the other malonate ester prodrugs and was far more effective at doing so than DMM.

The improved malonate ester prodrugs also reduced ROS production by succinate-driven RET in heart mitochondria (Fig. 5f). Whilst MHF reduced ROS production more than MAM, less malonate was detected in the mitochondria, suggesting that this difference is not through SDH inhibition, rather other effects such as off-target inhibition or mitochondrial toxicity.

### Malonate Ester Prodrugs Improve Cellular Malonate Delivery

**Fig. 6:**
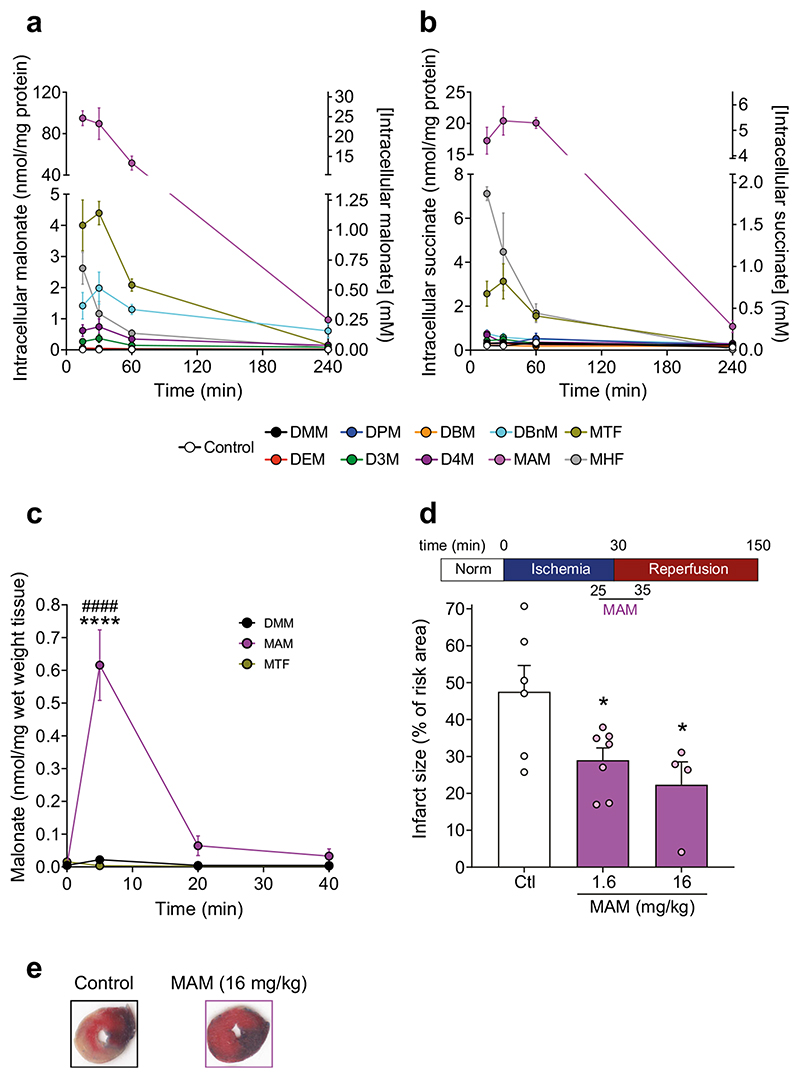
Improved malonate esters deliver far more malonate to cells in vitro and in vivo and is cardioprotective. **a-b** C2C12 mouse myoblasts were incubated with malonate diesters (250 μM) or 0.1% DMSO for 15, 30, 60 or 240 min before measuring intracellular malonate (**a**) and succinate (**b**) by LC-MS/MS (mean ± SEM, *n* = 3). **c** C57BL/6 J mice were injected with a bolus of DMM, MAM orMTF (160 mg/kg) in 0.9% saline +0.1% DMSO via tail vein injection. The mice were sacrificed either 5, 20 or 40 min after injection and malonate levels in the heart measured by LC-MS/MS (mean ± SEM, *n* = 3–5). Statistical significance was assessed by two-way ANOVA with Tukey’s correction for multiple comparisons where ****p < 0.0001 (compared with DMM) and *####p* <0.0001 (compared with MTF). **d**, **e** MAM is protective against cardiac I/R injury when given upon reperfusion. C57BL/6 J mice were subjected to 30 min of ischemia by occlusion of the left anterior, descending coronary artery, before 120 min of reperfusion. Either control (0.9% saline) or MAM were infused during reperfusion at 1.6 or 16 mg/kg. Results are presented as infarct size as a percentage of the area at risk, calculated by TTC staining. Representative heart images depicted in (**e**). Statistical significance was assessed by one-way ANOVA with Dunnett’s correction for multiple comparisons, where *p < 0.05 (compared with control (Ctl))

To see if these malonate ester prodrugs have the potential to rapidly release malonate within cells, we incubated them with cell lines over time and compared malonate generation with that of DMM. C2C12 mouse myoblasts were incubated with the malonate diesters and the malonate and succinate levels in the cell pellet and extracellular supernatant measured by LC-MS/MS. We found that incubation with MAM delivered considerably more malonate (> 20-fold) to the cell than any other compound (Fig. 6a and Supplementary Fig. 3) and delivered > 100-fold more malonate to the cell than DMM. The malonate esters released more malonate in the supernatant than the non-enzymatic hydrolysis incubations, suggesting that some malonate produced intracellularly could leave the cell (Supplementary Fig. 4a). The decrease in malonate in the cell over time likely reflects the endogenous metabolism of the compound. Malonate is readily conjugated to CoA using ATP by acyl-CoA synthetase family member 3 (ACSF3) in mitochondria [42]. Malonyl-CoA is subsequently decarboxylated by malonyl-CoA decarboxylase (MLCYD) to acetyl-CoA, and as this enzyme has both mitochondrial and cytosolic isoforms [43, 44], malonate can be readily metabolised. Thus, despite achieving high malonate levels in the cell and inhibiting SDH initially, it is rapidly metabolised by endogenous pathways, enabling SDH activity to resume.

Interestingly, cellular succinate levels were also significantly elevated in MAM treated cells (Fig. 6b), confirming mitochondrial delivery of malonate and inhibition of SDH. Here, the malonate delivered by MAM inhibits SDH, preventing the oxidation of succinate and leading to its extensive accumulation and transport out of the cell (Supplementary Fig. 4b). Succinate levels in DMM treated cells were not significantly different from control cells, suggesting that the levels of malonate achieved here are low. Importantly, similar results were seen in different cell types, and also when FBS was included in the incubations, despite its esterase activity and protein binding, which mimics that of the blood stream (Supplementary Fig. 5) [45, 46]. As MAM achieved the best delivery of malonate in cells, with acceptable cellular toxicity, as measured by LDH release (Supplementary Fig. 6), it was taken forward to assess in vivo malonate delivery and compared against DMM.

### MAM Delivers Malonate Rapidly to the Heart

To test the delivery of malonate in tissues in vivo, C57BL/6 J mice were tail vein injected with a bolus of either DMM, MAM or MTF (160 mg/kg in 100 μl) and the malonate levels in the heart assessed (Fig. 6c). MAM delivered significantly more malonate to the myocardium than DMM or MTF, which was also rapidly lost from the heart over time presumably due to metabolism to malonyl-CoA (Fig. 6c). The kinetics of malonate delivery to the heart by MAM suggest a useful profile for the prevention of cardiac I/R injury, whereby a transient inhibition of SDH is achieved during early reperfusion but with subsequent restoration of normal mitochondrial function.

### MAM Is Cardioprotective when Administered at Reperfusion

We next tested the cardioprotective effect of MAM in a murine left anterior descending (LAD) coronary artery occlusion model of MI, whereby the artery is occluded for 30 min and subsequently reperfused with or without an intervention (Fig. 6d). Whilst 160 mg/kg of MAM was toxic to the animals undergoing the LAD model (data not shown, we note there was no obvious distress to the animals prior to sudden death which suggested that the toxicity was not due to MAM breakdown products directly but was a complicated interaction with anesthesia), administration of either a 10- or 100-fold lower dose (16 or 1.6 mg/kg) of MAM during early reperfusion was significantly cardioprotective compared with relevant control animals and to DMM at the higher dose (Fig. 6d, e). Therefore, by developing a rapidly cleaved malonate ester, we were able to deliver sufficient malonate when administered at reperfusion to inhibit SDH, slow down the oxidation of succinate and prevent ROS production by RET.

## Discussion

DMM has been extensively used as a cell permeable SDH inhibitor, particularly in elucidating the mechanism of succinate’s involvement in I/R injury. Whilst preventing succinate accumulation with DMM is protective, clinically relevant agents for MI are limited to being administered concurrent to reperfusion. DMM hydrolysed slowly in the in vitro setting and thus confirmed why DMM was not protective when administered at reperfusion. By developing more rapidly hydrolysed malonate esters, these were able to deliver far more malonate to cells and in vivo, with MAM the most effective.

The two approaches taken to improve malonate delivery suggest that both drug hydrophobicity and rate of hydrolysis were important. As longer dialkyl esters could also deliver more malonate than DMM, it is likely that the favourable diffusion across the hydrophobic biological membranes led to increased potential for hydrolysis and subsequent intracellular malonate delivery. In addition, as MAM was both as hydrophobic as, and more rapidly hydrolysed than, DMM, this combination of molecular properties was clearly favourable for the delivery of small molecule dicarboxylates to the cell. This screening process also highlights the importance of careful prodrug ester selection, as this is critical for the development and efficacy of the intended compound.

The kinetics of malonate release from MAM may also be highly advantageous as an intervention for I/R injury. Malonate is released very rapidly into the heart tissue in the first minutes after administration of MAM, inhibiting SDH and preventing the pathological production of ROS during reperfusion. Whilst SDH is inhibited by malonate, normal mitochondrial function would resume and the ATP/ADP ratio restored. Malonate is subsequently metabolised, releasing the block on SDH and thus providing a transient effect during the therapeutic window. This action of MAM would minimize drug exposure and continual inhibition, thus reducing the possibility of off target and deleterious effects.

The cardioprotective effect of MAM further confirms the role of SDH as a critical enzyme in the pathology associated with I/R injury. We have now shown that targeting SDH either before ischemia using DMM or at reperfusion using MAM leads to a reduction in infarct size upon reperfusion. Both approaches alter the metabolism of succinate, either its production or oxidation by SDH, thus highlighting its critical role in I/R injury and an important target for translation. Moreover, the cardioprotective effect of MAM was achieved at both 16 and 1.6 mg/kg, much lower concentrations than required by DMM. Despite not changing the potency of the SDH inhibitor, the extent of delivery of the active compound is critical. By enabling a large reduction in the total dose to be delivered by using MAM, this also not only aids in avoiding side effects but also means an appropriate dose could be delivered to large animals and humans. As large infusion volumes for MI patients are unfavourable, the lower concentration of MAM required would also enable delivery of malonate in a minimal volume.

Whilst the malonate levels in the heart that led to cardioprotection in vivo were not measured, the protection is dependent on the levels of malonate delivered to the tissue. When the lowest dose of MAM (1.6 mg/kg) was infused over an extended period of time during reperfusion, this was not protective (data not shown). Due to the rapid hydrolysis and clearance of malonate from MAM, this will have led to a lower concentration of malonate achieved in the heart tissue, at a level where SDH is not efficiently inhibited. Therefore, this suggests that the cardioprotective effect of MAM is dependent on the level of malonate delivered to the heart. Overall, more extensive studies on the malonate levels in the heart at reperfusion and long-term protection are warranted to confirm its translational potential.

## Conclusion

Targeting SDH in ameliorating I/R injury has gained interest due to its mechanistic importance in both succinate accumulation during ischemia and subsequent oxidation upon reperfusion. Whilst DMM has been shown to inhibit the accumulation of succinate and prevent I/R injury in vivo, it was ineffective when administered at reperfusion, where clinically, it is of interest due to the appeal of concurrent administration with PPCI. Here, we screened a range of malonate ester prodrugs and found that caging malonate within an improved esterase substrate such as MAM led to significantly improved malonate delivery both in vitro and in vivo. Moreover, MAM also provided cardioprotection when administered at reperfusion; thus, we have developed a clinically relevant therapeutic that could be administered to MI patients during PPCI. However, considerably more work will be required to assess how well MAM protects the heart from the long-term cardiac dysfunction and scarring caused by IR injury.

Furthermore, by expanding the palette of malonate esters available, the correct malonate-based therapeutic for the ischemia-related pathology can be chosen. Whilst MAM may be optimal for MI, other esters that release malonate more slowly may be more optimal for use in instances such as organ transplantation, where the treatment window is predictable and extended. Overall, the development of SDH-targeted therapeutics presents a promising strategy for the treatment of I/R injury.

## Supplementary Material

Supplementary Material

## Data Availability

The data corresponding organic synthesis is available at https://doi.org/10.5525/gla.researchdata.1007. Other data supporting the findings in this study will be made available upon reasonable request to the corresponding authors.

## Abbreviations

ROS: reactive oxygen species
I/R: ischemia/reperfusion
LAD: left anterior descending coronary artery
MI: myocardial infarction
PPCI: primary percutaneous coronary intervention
CAC: citric acid cycle
CoQ: coenzyme Q
Δp: proton motive force
RET: reverse electron transport
LC-MS/MS: liquid chromatography-tandem mass spectrometry
DMM: dimethyl malonate
DEM: diethyl malonate
DPM: diisopropyl malonate
D3M: dipropyl malonate
DBM: di-tert-butyl malonate
D4M: dibutyl malonate
DBnM: dibenzyl malonate
MAM: diacetoxymethyl malonate
MTF: ditrifluoroethyl malonate
MHF: dihexafluoropropyl malonate
PLE: porcine liver esterase
