# Supplementary material for: Ester Prodrugs of Malonate with Enhanced Intracellular Delivery Protect Against Cardiac Ischemia-Reperfusion Injury In Vivo
Source: Cardiovasc Drugs Ther. Author manuscript; Available in PMC 2022 Feb 1. (PMC8770414; doi:10.1007/s10557-020-07033-6)
Supplement: Supplementary Material [file EMS128238-supplement-Supplementary_Material.docx]

**Electronic Supplementary Material**

**Ester Prodrugs of Malonate with Enhanced Intracellular Delivery Protect Against Cardiac Ischemia-Reperfusion Injury *In Vivo***

**Hiran A. Prag^1,2^ • Laura Pala^3^ • Duvaraka Kula-Alwar^2^ • John F. Mulvey^2^ • Du Luping^4^ • Timothy E. Beach^5^ • Lee M. Booty^1^ • Andrew R. Hall^1^ • Angela Logan^1^ • Volha Sauchanka^2^ • Stuart T. Caldwell^3^ • Ellen L. Robb^1^ • Andrew M. James^1^ • Zhelong Xu^4^• Kourosh Saeb-Parsy^5^ • Richard C. Hartley^3^ • Michael P. Murphy^1,2*^ • Thomas Krieg^2*^**

^1^MRC Mitochondrial Biology Unit, University of Cambridge, Cambridge Biomedical Campus, CB2 0XY, UK

^2^Department of Medicine, University of Cambridge, Cambridge, CB2 0QQ, UK

^3^School of Chemistry, University of Glasgow, Glasgow, G12 8QQ, UK

^4^Tianjin Medical University, Tianjin 300070, China

^5^Department of Surgery, University of Cambridge and NIHR Cambridge Biomedical Research Centre, Cambridge, CB2 0QQ, UK

Correspondence to: Thomas Krieg ([tk382@medschl.cam.ac.uk](mailto:tk382@medschl.cam.ac.uk)) or Michael P. Murphy ([mpm@mrc-mbu.cam.ac.uk](mailto:mpm@mrc-mbu.cam.ac.uk)).

**
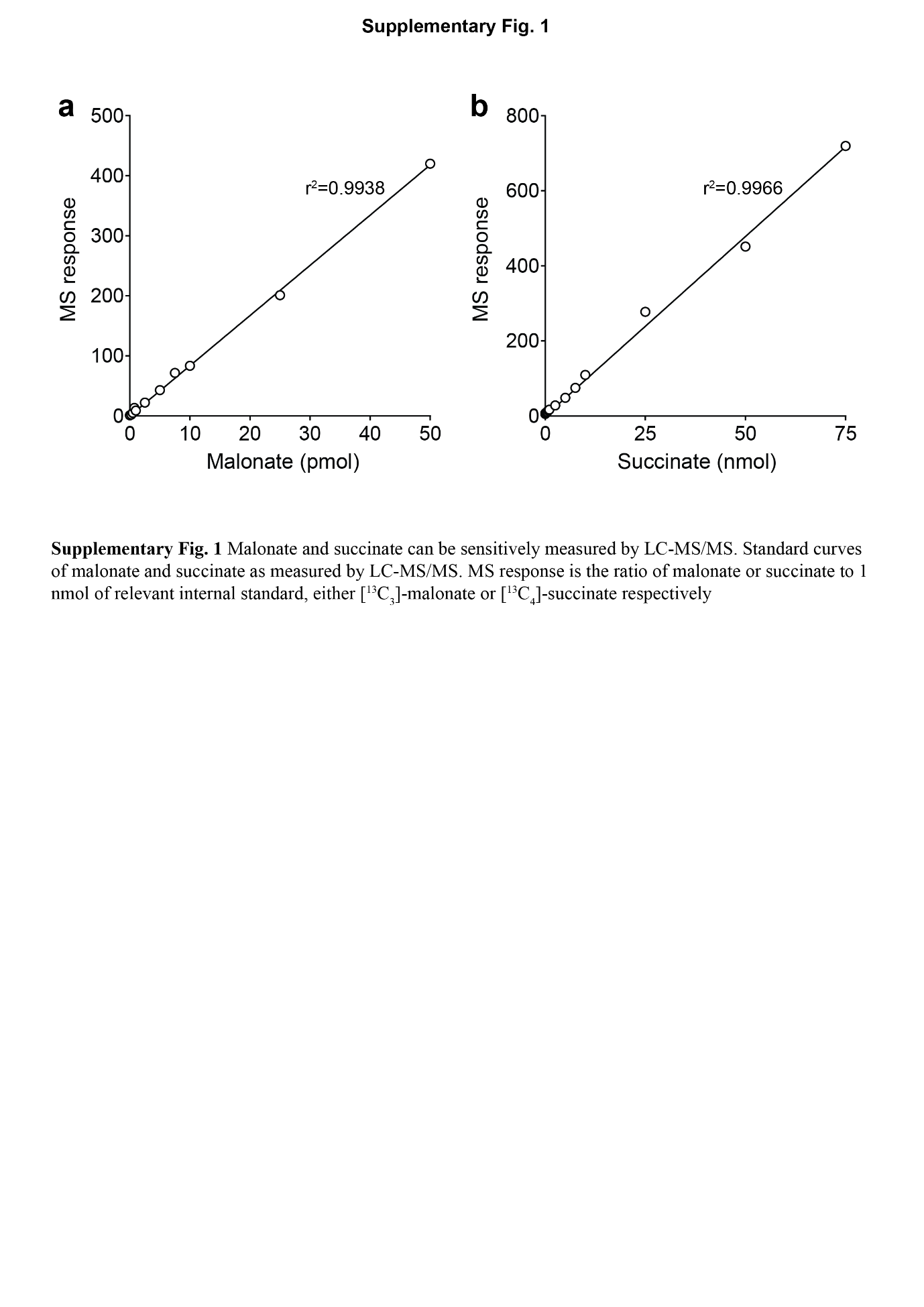
**

**Supplementary Fig. 1** Malonate and succinate can be sensitively measured by LC-MS/MS. Standard curves of malonate and succinate as measured by LC-MS/MS. MS response is the ratio of malonate or succinate to 1 nmol of relevant internal standard, either [^13^C_3_]-malonate or [^13^C_4_]-succinate respectively


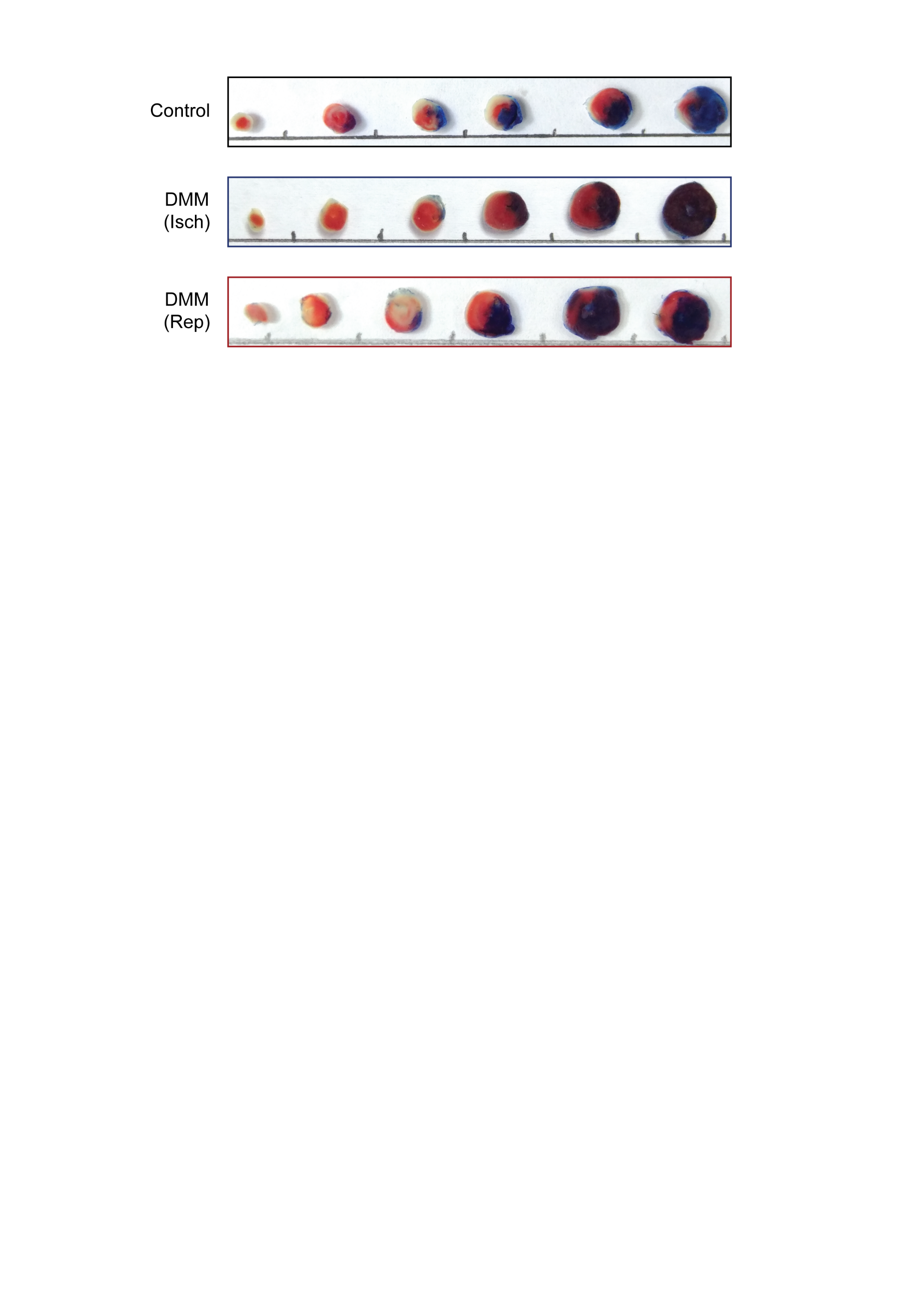


**Supplementary Fig. 2** Protection against I/R injury by DMM. Sections from a representative heart in experiments where infarct size as quantified in Fig. 2a**.** C57BL/6J mice were subjected to 30 min LAD occlusion before 120 min reperfusion and infarct assessed by TTC staining. 0.9% saline or DMM (160 mg/kg total dose) dissolved in 0.9% saline were administered i.v. either before ischemia (Isch) or at reperfusion (Rep)

**
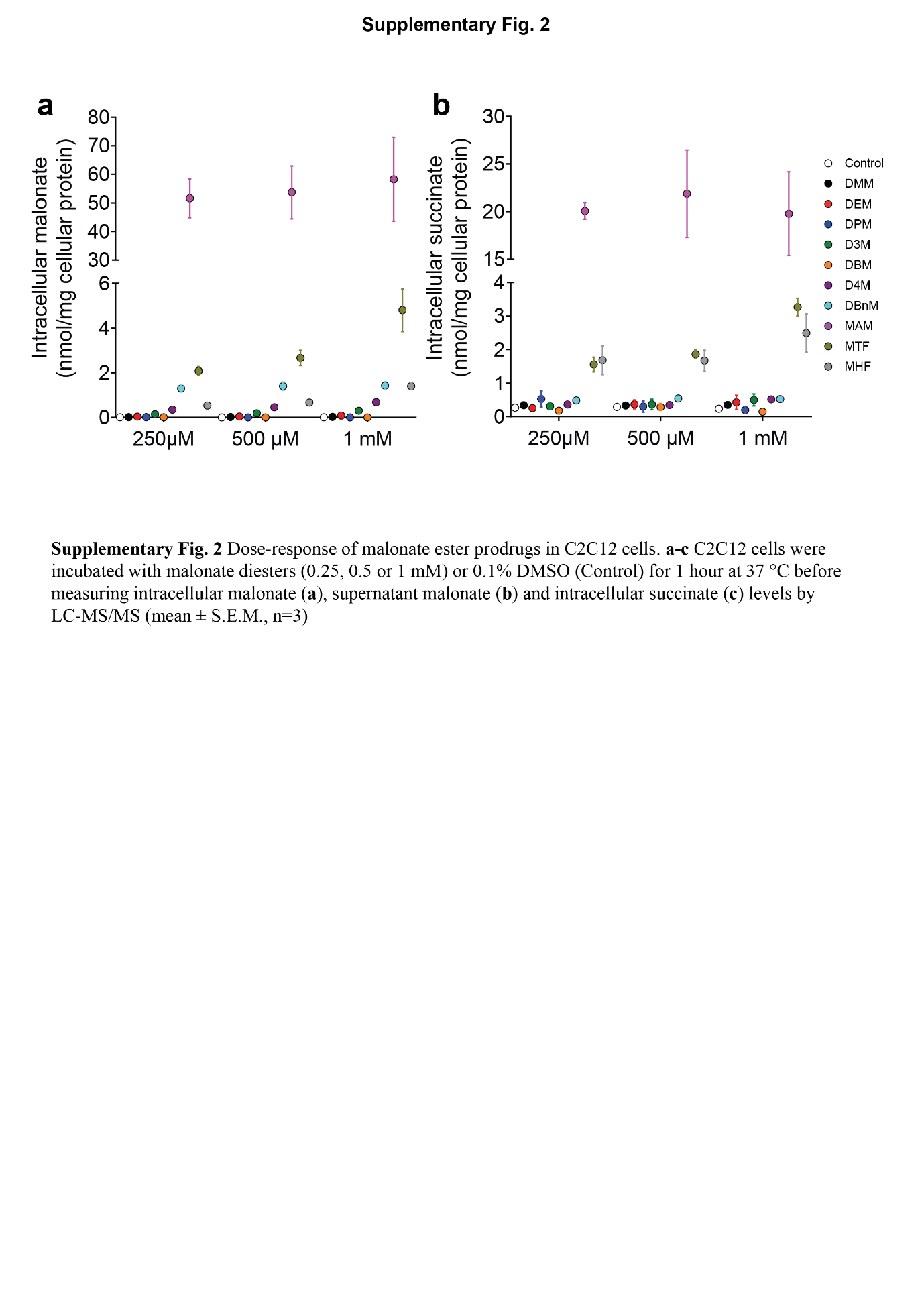
**

**Supplementary Fig. 3** Dose-response of malonate ester prodrugs in C2C12 cells. **a-c** C2C12 cells were incubated with malonate diesters (0.25, 0.5 or 1 mM) or 0.1% DMSO (Control) for 1 hour at 37 °C before measuring intracellular malonate **(a)**, supernatant malonate **(b)** and intracellular succinate **(c)** levels by LC-MS/MS (mean ± S.E.M., n=3)

**
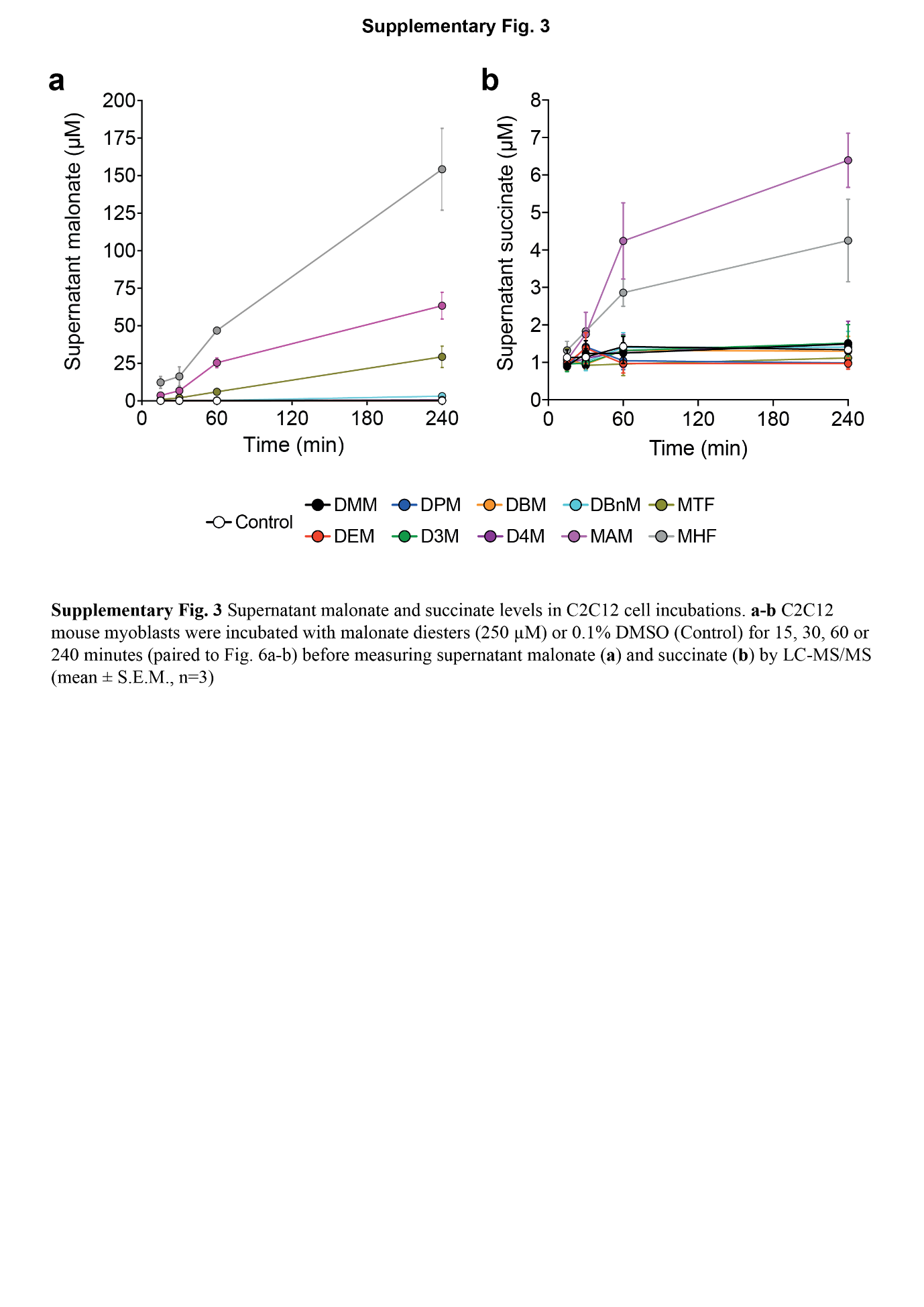
**

**Supplementary Fig. 4** Supernatant malonate and succinate levels in C2C12 cell incubations. **a-b** C2C12 mouse myoblasts were incubated with malonate diesters (250 µM) or 0.1% DMSO (Control) for 15, 30, 60 or 240 minutes (paired to Fig. 6a-b) before measuring supernatant malonate **(a)** and succinate **(b)** by LC-MS/MS (mean ± S.E.M., n=3)

**
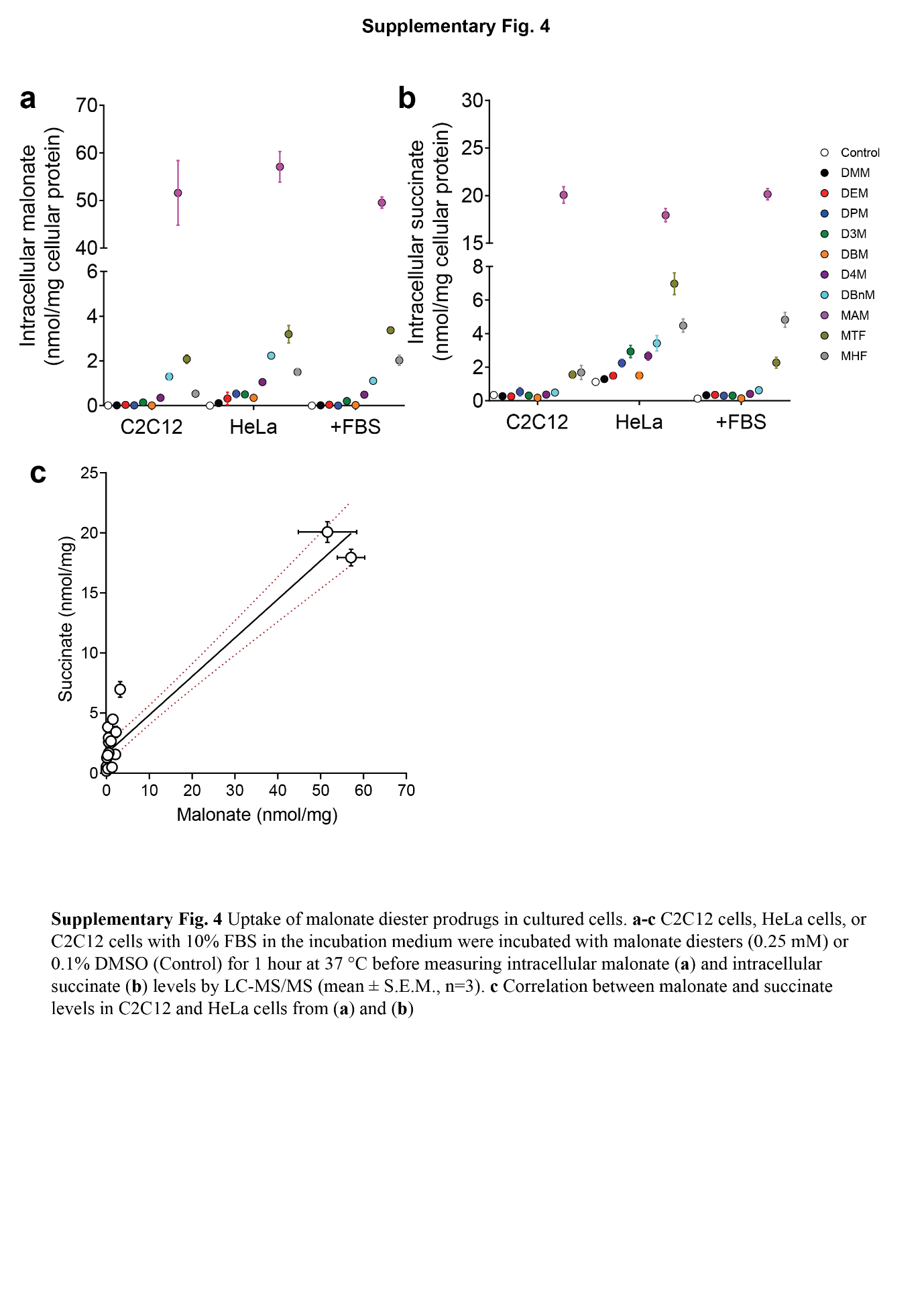
**

**Supplementary Fig. 5** Uptake of malonate diester prodrugs in cultured cells. **a-c** C2C12 cells, HeLa cells, or C2C12 cells with 10% FBS in the incubation medium were incubated with malonate diesters (0.25 mM) or 0.1% DMSO (Control) for 1 hour at 37 °C before measuring intracellular malonate **(a)** and intracellular succinate **(b)** levels by LC-MS/MS (mean ± S.E.M., n=3). **c** Correlation between malonate and succinate levels in C2C12 and HeLa cells from **(a)** and **(b)**

**
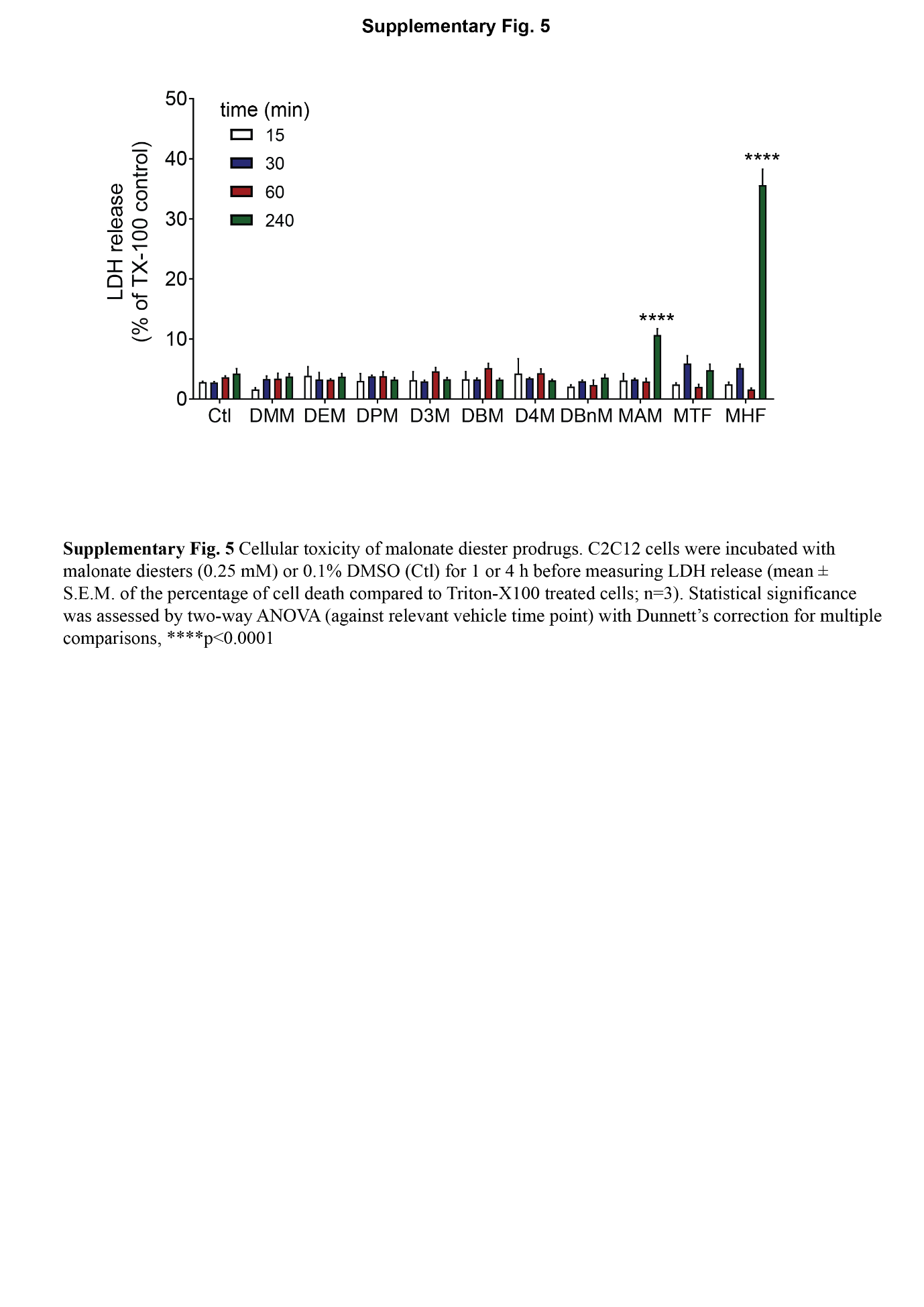
**

**Supplementary Fig. 6** Cellular toxicity of malonate diester prodrugs. C2C12 cells were incubated with malonate diesters (0.25 mM) or 0.1% DMSO (Ctl) for 1 or 4 h before measuring LDH release (mean ± S.E.M. of the percentage of cell death compared to Triton-X100 treated cells; n=3). Statistical significance was assessed by two-way ANOVA (against relevant vehicle time point) with Dunnett’s correction for multiple comparisons, ****p<0.0001

**Supplementary Methods**

**Cellular toxicity.** C2C12 mouse myoblasts were plated in 96-well plates (10,000 cells/well) and adhered overnight in a humidified incubator at 37 °C and 5% CO2. The following day, media was replaced with phenol red and FBS-free DMEM media with the indicated treatments or vehicle DMSO control using the same conditions as that for the cell uptake studies. Compound titration of 250 µM, 500 µM or 1 mM were incubated for 1 hour and time courses of 250 µM were incubated for 15, 30, 60 or 240 minutes. Substance controls without cells and Triton X-100 were used as low and high controls. After the appropriate incubation periods, cells were centrifuged (250 x g, 10 min, 4 °C) and 100 µl supernatant removed and assessed for LDH release using cytotoxicity detection kit (Roche, UK) according to manufacturer's instructions.

**Chemical Syntheses**

All reactions under an inert atmosphere were carried out using flame-dried glassware and solvents were added *via* syringe. Reagents were obtained from commercial suppliers and used without further purification. Dry solvents were collected from a Puresolv solvent purification system or obtained from commercial suppliers. ^1^H NMR spectra were obtained using Bucker-ACS 60 spectrometer operating at 400 and 500 MHz, ^13^C NMR spectra at 101 and 126 MHz, ^31^P NMR spectra at 162 and 202 MHz, ^19^F spectra at 377 and 471 MHz. Signal splitting patterns were described as: singlet (s), doublet (d), triplet (t), quartet (q), quintet (qn), sextuplet (sx), septuplet (sept), multiplet (m), broad singlet (broad s), or any combination of the above. All coupling constants were recorded in Hz. In ^1^H NMR spectra tentative assignment was done on the base of the chemical shift, definitive on the base on COSY (when made). DEPT was used to assign the signals in ^13^C NMR spectra as C, CH, CH_2_ and CH_3_. 2D techniques including COSY and HSQC were used to help assignment. Deuterated solvents contained trimethylsilane (TMS) as a reference compound. All spectra were assigned using following reference solvent peaks: CDCl_3_ (7.26 ppm for ^1^H NMR; 77.16 ppm for ^13^C NMR), CD_3_CN (1.94 ppm for ^1^H NMR; 118.26 ppm for ^13^C NMR), DMSO-*d_6_* (2.50 ppm for ^1^H NMR, 39.52 ppm for ^13^C NMR). LRMS (ESI^+^) and HRMS (ESI^+^) spectra were collected on a Bruker MicroTOF-Q, EI spectra were collected on a Jeol JMS700 (MStation) spectrometer. IR spectra were obtained using Shimadzu FTIR-8400S. Reactions were monitored by thin layer chromatography (TLC) performed on aluminium sheets pre-coated with silica gel (Merck or Fluorchem Silica Gel 60 F254) and visualisation was performed using UV light (λ_max_ = 254 or 365 nm) or by staining with a potassium permanganate solution dip. Purification of products was carried out by re-crystallization, distillation under vacuum or Biotage® Isolera^TM^ One Flash Chromatography system using Biotage® SNAP Ultra silica gel cartridges.

# *Bis(acetoxymethyl) malonate (MAM)*

Following the procedure of Bao *et al* [1], malonic acid (1.00 g, 9.61 mmol, 1.0 eq) and *N*,*N*-diisopropylethylamine (2.1 mL, 21 mmol, 2.2 eq) were dissolved in dry acetonitrile (10 mL) while stirring under an atmosphere of argon and bromomethyl acetate (2.1 mL, 21 mmol, 2.2 eq) was added. The solution was stirred at RT for 24 h. The solvent was evaporated under reduced pressure and the crude was purified by column chromatography [SiO_2_, hexane-ethyl acetate (7:3)] to yield the tetraester as a colourless oil (1.58 g, 6.37 mmol, 66%). R*_f_* [SiO_2_, hexane-ethyl acetate (7:3)] = 0.26. δ_H_ (400 MHz, CDCl_3_): 5.78 (4H, s, OCH_2_O), 3.48 (2H, s, CH_2_CO), 2.13 (6H, s, CH_3_). δ_C_ (101 MHz, CDCl_3_): 169.58 (C), 164.79 (C), 79.71 (CH_2_), 40.94 (CH_2_), 20.76 (CH_3_). LRMS (ESI^+^): 271 [(M+Na)^+^, 100%]. HRMS (ESI^+^): 271.0422. C_9_H_12_NaO_8_^+^ requires (M+Na)^+^, 271.0424. The NMR data agree with literature data [1].

# *Bis(trifluoroethyl) malonate (MTF)*

Malonyl chloride (1.00 mL, 10.3 mmol, 1.0 eq) was dissolved in 2.0 mL of 2,2,2-trifluoroethanol under an atmosphere of argon, then triethylamine (320 μL, 2.26 mmol, 2.2 eq) was added dropwise. The reaction mixture was stirred for 24 h at RT under an atmosphere of argon, quenched with 1 M hydrochloric acid (20 mL) and extracted with dichloromethane (3 × 20 mL). The organics were washed with brine (2 × 20 mL), dried over magnesium sulfate, filtered and the solvent was evaporated under reduced pressure. The diester was distilled under reduced pressure to yield a colourless oil (1.68 g, 6.27 mmol, 61%). δ_H_ (400 MHz, CDCl_3_): 4.55 (4H, q, *J* = 8.2, **C**H_2_CF_3_), 3.61 (2H, s, **C**H_2_CO_2_). δ_C_ (101 MHz, CDCl_3_): 164.22 (C), 122.66 (q, *J* = 277.1 Hz, C), 61.32 (q, *J* = 37.2 Hz, CH_2_), 40.25 (CH_2_). δ_F_ (377 MHz, CDCl_3_): −73.83 (s, F-H decoupled, CF_3_). LRMS (ESI^+^): 291 [(M+Na)^+^, 100%]. HRMS (ESI^+^): 291.0056. C_7_H_6_F_6_NaO_4_^+^ requires (M+Na)^+^, 291.0062. The NMR data agree with literature data [2].

# *Di(1,1,1,3,3,3-hexafluoro-2-propyl) malonate (MHF)*

Malonyl chloride (1.00 mL, 10.3 mmol) was dissolved in 1,1,1,3,3,3-hexafluoro-2-propanol (5.0 mL) under an atmosphere of argon. The reaction was heated under reflux for 16 h. After cooling at RT, the solvent was removed under reduced pressure and the product was distilled under reduced pressure to yield the diester as a yellow oil (1.55 g, 3.84 mmol, 37%). *ν*_max_ (ATR): 2972 (CH), 2928 (CH) 1792 (C=O), 1732 (C=O), 1559 (Ph) cm^-1^. δ_H_ (500 MHz, CDCl_3_): 5.79 (2H, sept, *J* = 5.8 Hz, CH), 3.81 (2H, s, CH_2_). δ_C_ (126 MHz, CDCl_3_): 161.95 (C), 120.14 (qq, *J* = 2.7, 282.2 Hz, C), 67.67 (sept, *J* = 35.3 Hz, CH), 39.52 (CH_2_). δ_F_ (470 MHz, CDCl_3_): −73.23 (d, *J* = 5.9 Hz, CF_3_). HRMS (CI^+^): 404.9996. C_9_H_5_F_12_O_4_^+^ requires (M+H)^+^, 404.9996.

**Supplementary references**

1. Bao X, Zhao Q, Yang T, Fung YME, Li XD. A chemical probe for lysine malonylation. Angew Chemie Int Ed. 2013;52:4883–6.

2. de Nanteuil F, Loup J, Waser J. Catalytic Friedel–Crafts Reaction of Aminocyclopropanes. Org Lett. 2013;15:3738–41.
